# Measuring Mental Health Service Accessibility for Indigenous Populations: a Systematic Review

**DOI:** 10.1007/s40615-023-01899-6

**Published:** 2024-01-25

**Authors:** Lihong Zhang, Xiang-Yu Hou, Yan Liu

**Affiliations:** 1 School of the Environment, The University of Queensland, Brisbane, Queensland Australia; 2 Poche Centre for Indigenous Health, The University of Queensland, Brisbane, Queensland Australia

**Keywords:** Mental health services, Accessibility, Indigenous populations, Spatial inequality, Systematic review, 91D20

## Abstract

Indigenous populations have experienced inequality of accessing mental health services compared with their non-Indigenous counterparts, although the way of measuring mental health service accessibility for Indigenous populations is unclear. This systematic review examines measures of mental health service accessibility for Indigenous people, including the diversity of mental health services that are available to them and the barriers to accessing mental healthcare. Using a systematic search procedure, we identified 27 studies that explored Indigenous populations’ mental health service access. Our review shows that 18 studies used interview-based methods to explore how Indigenous people use mental health services, and only nine studies used quantitative methods to measure the uptake of mental health services. While advanced methods for quantifying geographical access to healthcare services are widely available, these methods have not been applied in the current literature to explore the potential access to mental health services by Indigenous populations. This is partially due to limited understanding of how Indigenous populations seek mental healthcare, barriers that prevent Indigenous people from accessing diverse types of mental health services, and scarcity of data that are available to researchers. Future research could focus on developing methods to support spatially explicit measuring of accessibility to mental health services for Indigenous populations.

## Introduction

Indigenous populations—around 480 million people worldwide—persistently experience poorer mental health outcomes compared to their non-Indigenous counterparts [1, 2]. According to the World Health Organisation (WHO), Indigenous populations are defined as those who are descendants of original inhabitants of a region prior to the establishment of modern states and borders; these populations typically reside in defined geographical territories and self-identify as members of cultural groups distinct from the mainstream society [3]. The enduring impact of colonisation, intergenerational trauma, and socioeconomic disadvantages has led to a relatively low health status among Indigenous peoples, such as the *Aboriginal and Torres Strait Islanders* in Australia [4], *Mãori* in New Zealand [5], *Inuit, Indians, and Metis people* in Canada [2], and *African-Americans, Latinos, Native Americans, and Asian and Pacific Islanders* in the USA [6]. On the other hand, Indigenous people are rendered to be more vulnerable to mental illness [7], which has been estimated as the second highest contributor to disease burden in the Indigenous populations [8]. This situation is accompanied by a disproportionately high level of unmet needs and a low level of access to mental health services [9], which is further exacerbated by the institutional racism experienced in seeking mental health services [7].

To improve Indigenous populations’ mental health status, it is important to enhance their access to mental health services [10]. With a growing body of literature examining the disparities in mental health service usage for racial-ethnic communities including Indigenous groups [11, 12], enhancing access to mental health services for Indigenous peoples has been suggested as effective interventions to eliminate such disparities [10, 13]. Existing studies have explored the access to mental healthcare from various dimensions, including those focusing on particular types of mental health services (e.g., mental healthcare in primary healthcare services [4]), particular Indigenous population cohorts (e.g., the youth [14]), inequalities in service utilisation [15], and optimising the pathways to healthcare [16]. These studies usually assess the access to mental healthcare qualitatively rather than measure explicitly spatial accessibility and identify areas with a shortage of mental health services [17, 18].

Empirically, *access to mental health services* is a mixed concept which could indicate either realised access (i.e., actual service utilisation) [19, 20], or potential access (i.e., service provision and distribution) [21]. Since accessing mental health services is the pre-condition for service utilisation, and service shortage could lead to low level of utilisation, it is important to explore the potential access to mental health services for Indigenous populations. The potential access to mental health services could involve individuals who need the services, the way individuals travel to access the services, and the types of services available to individuals [22]. Potential access can be measured as geographical accessibility, namely the extent of individuals’ potential access to available services within a specific geographical area [23].

Studies on measuring the geographical accessibility to health services (including mental health services) have advanced over the past decade, and the approaches used vary according to (i) types of services by providers (e.g., integrated clinics in communities) [24]; (ii) elements of travelling to access health services (e.g., travel distance, time, and cost) [21]; (iii) ways in which services are delivered (e.g., office-based practice and outpatient health treatment facility) [25]; (iv) people’s perceptions (i.e., perceived accessibility) [26]; and (v) appeals of service providers [27]. The use of spatial techniques such as Geographic Information System (GIS) has also progressed from simple mapping to spatially explicit modelling of accessibility to uncover how individuals access health services through the built environment they live in, with outcomes contributing to assisting government authorities in identifying disparities in the provision of health services [28, 29]. Given the different needs of health services by people with different demographic and socioeconomic characteristics, it has attracted scholarly interest to measuring geographical accessibility to health services for various population groups (e.g., by ages [22] and income [25]). Methodological advancement has been made in measuring geographical access to health services in recent years, and the outcome being used to support decision-making relating to health service allocation [30]. However, significant disparities exist in the measured geographical accessibility using different methods, which limits its potential in supporting decision-making. Nevertheless, it remains unexplored in considering people’s Indigenous status and how they gain access to mental health services.

For Indigenous people, the variability and availability of services that are culturally appropriate to use and meet their population-specific needs would influence their utilisation of the services [15]. Thus, understanding how Indigenous populations seek mental healthcare and the obstacles impeding their access to different types of mental health services is crucial for comprehending and measuring the geographical accessibility to mental health services for them. It is essential to collect data quantifying mental health service supply and demand of such services considering barriers for Indigenous people, in order to develop an accessibility measure that can be generalised to different Indigenous populations, can be replicated with a high degree of precision, and can capture changes across different conditions in a quantifiable manner [31]. The availability of such data can be influenced by the actualities of service provision and the barriers faced by Indigenous individuals in accessing mental health services, necessitating further examination.

To tackle the disparity in mental health service accessibility between Indigenous and non-Indigenous peoples, it is crucial to understand how accessibility to mental health services is measured for Indigenous people, while currently, measures of this accessibility remain largely uncharted. This systematic review aims to synthesise existing scholarship on how accessibility to mental health services for Indigenous people are studied, identify knowledge gaps, and pave pathways for future research to inform planning and policymaking for addressing the inequities in accessing mental health services for Indigenous groups.

## Method

Our systematic review approach follows the guidelines set forth in the PRISMA Statement for Reporting Systematic Reviews [32]. We employed a mixed-method approach for the review [33], combining empirical findings with relevant theoretical and policy advancements. This method enables us to combine the rigorous systematic review approaches using keyword choice and source identification with the adaptability of conventional literature reviews. In the current study, no review protocol was utilized.

**Fig. 1:**
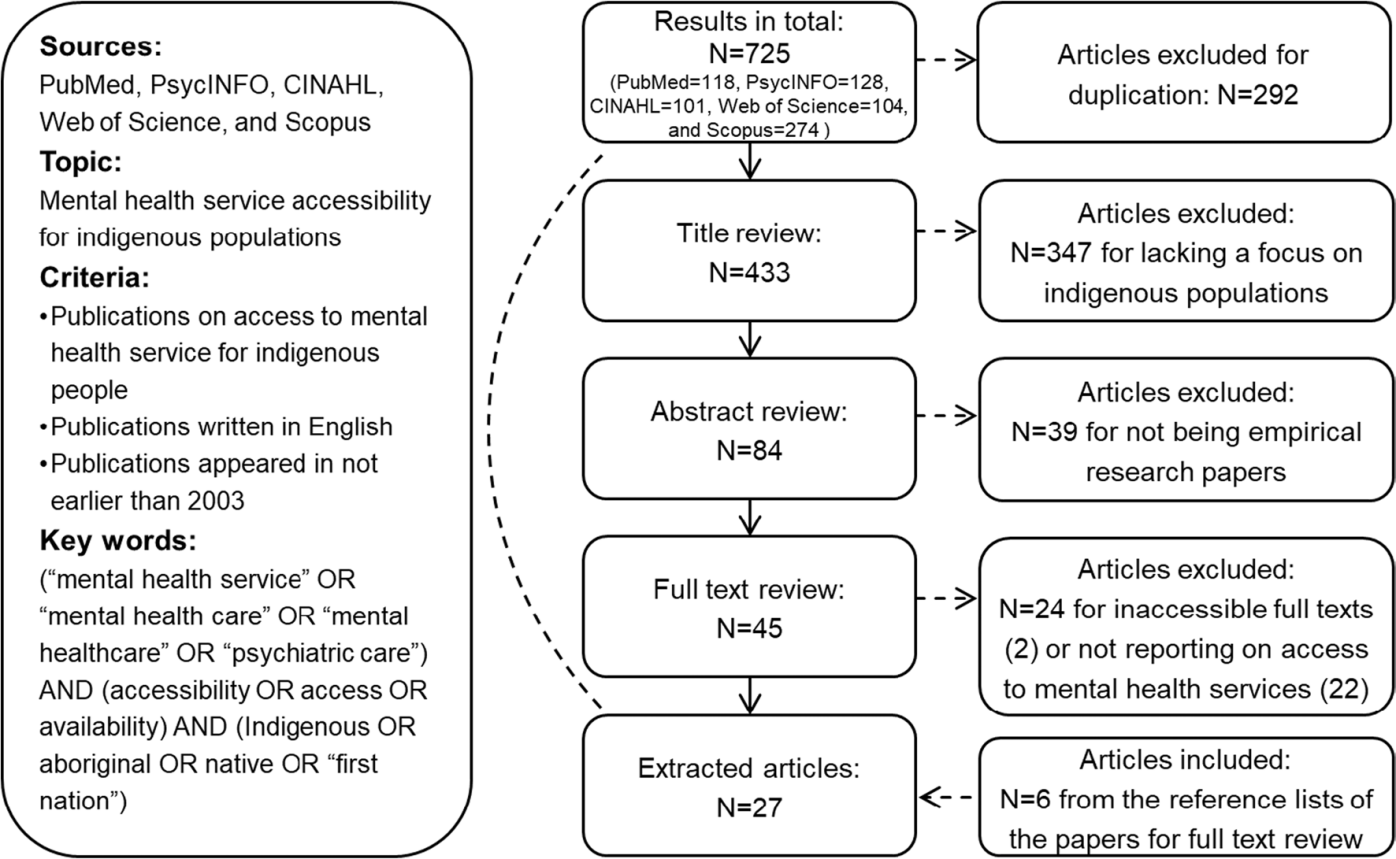
Selection of literature for our systematic review using the PRISMA flow diagram

The articles included in this study were obtained from five databases: Web of Science, PubMed, PsycINFO, CINAHL, and Scopus. We employed a comprehensive set of keywords to encompass various terms related to Indigenous populations’ mental health service studies. These terms were derived from our knowledge of the research field and were identified during the systematic review process. Our primary search term was “(mental health service) AND accessibility AND Indigenous”. We also used variations such as “mental health care”, “mental healthcare”, and “psychiatric care” in place of “mental health service”. Additionally, we substituted “accessibility” with “access”, “availability”, and “Indigenous” with “aboriginal”, “native”, and “first nations”. To ensure comprehensive coverage, we thoroughly examined the reference lists of included articles. Our initial search for published studies was conducted on 29 March 2023, and we concluded our literature collection on 31 July 2023. All articles we reviewed were published in English. Figure 1 illustrates the selection process (and outcome) for our systematic review using the PRISMA flow diagram.

Furthermore, policy documents for local situations arose during the literature reviewing process. While findings and practices from these documents were synthesised and discussed in some published articles, the policy documents themselves were not counted as academic literature in this review. Instead, these documents were considered as examples of empirical practices in mental health services, reflecting specific local contexts that may not represent all possible cases around the world.

## Results

Our search initially yielded 725 candidate documents. After removing 292 duplicates, titles, and abstracts of the remaining documents were screened for eligibility considering the focus on Indigenous populations and empirical research papers (i.e., excluding articles like clinical reports, public commentary, and magazines), which resulted in 45 articles. Then, full texts of these 45 articles were screened, leading to the exclusion of an additional 24 articles due to not reporting on access to mental health services (e.g., access was mentioned but not discussed due to their research topics of other aspects such as mental health treatments). The references used in the 45 articles included in the full-text screening were also reviewed, resulting in six additional studies being included in the final set and subsequently analysed. As a result, a total of 27 studies were selected for further review (see Fig. 1). The final selected studies were published between 1992 and 2023 and were conducted in countries including Canada, the USA, Australia, New Zealand, and a few countries in Europe (Table 1).

### Qualitative Exploration of Mental Health Service Accessibility for Indigenous People

Accessibility to mental health services for Indigenous people has been studied using qualitative methods, including interviews, narrative inquiries, questionnaire survey, thematic analysis, and critical analysis (Table 1). While these methods are primarily used for identifying help-seeking behaviours among Indigenous individuals to facilitate their utilisation of mental health services [34], access to mental health services for Indigenous people or a sub-group of them (e.g., youth) is a key component of these studies [14, 35]. However, the concept of *access* or *accessibility* in these studies is not specifically defined [36, 37] and has an underlying assumption that improving access to mental health services for Indigenous populations indicates more Indigenous people receiving mental health treatments [38]. Based on this assumption, the qualitative exploration of accessibility in the reviewed articles focus on Indigenous people’s experiences and perceptions of mental health services and take into account characteristics of mental health services that could influence individuals’ utilisation of the services. Various aspects of mental health services have been explored in existing literature, including mental healthcare providers (e.g., Indigenous community controlled services [39]), support for entering services (e.g., early intervention [17] and transport support [40]), and engagement with services (e.g., staffing problems [41] and service hours and waiting time [42]). There are some scholars approaching *access to mental health services* as a process that precedes the receipt of mental health treatment by Indigenous people [43, 44]. This process is composed of four stages, including (1) recognising the problem, (2) deciding to seek help, (3) deciding that the service to be received will be helpful, and (4) contacting the service provider (e.g., locating an accessible mental health service facility, selecting a proper health practitioner to seek treatment, and physically accessing the service facility) [43, 44]. The factors that can impact on this access process for Indigenous people include sociodemographic characteristics (e.g., gender [45], age [34], income [34]) and other individual characteristics (e.g., disabilities [46]).

Further, studies using qualitative method specify the accessibility by focusing on certain types of mental health services, such as the mainstream mental health service [43] and Indigenous community-controlled health service [40]. Marginalised groups in Indigenous populations, including gender minority [14, 45], those living in rural and remote areas [47], those with socioeconomic disadvantages (e.g., poverty) [34], those having substance use issues [15], and those with hearing impaired [46], are getting a growing scholarly attention in terms of defining access to mental health services catering to their needs. Treatments and health outcomes along with the settings of the service environments are also explored to enhance the accessibility to mental health services for the focused groups [17, 48]. The focused groups in different studies may have overlap with each other (e.g., socioeconomic disadvantageous groups and those having substance use issues [14, 15]), which makes summarising the findings of all groups difficult. Nevertheless, the qualitative research points out that Indigenous people, along with sub-groups within them, have differentiated situations that should be comprehensively considered when measuring their accessibility to mental health services.

### Geographical Measures of Accessibility to Health Services Are Available but not Applied to Mental Health Services for Indigenous People

While various quantitative approaches have been proposed from a geographical perspective and applied to measure access to health services, including mental healthcare, for population sub-groups, these methods have not yet been explicitly applied to measure Indigenous populations’ access to mental health services [25, 28, 49]. Some reviewed articles used quantitative methods such as logistic regressions to examine the uptake, population reach and its provider agency-level predictors, and outcomes of mental healthcare services provided to Indigenous people (Table 1). These studies involve the collection and analysis of numerical data on the utilisation of general or specific mental health services to understand and describe patterns of how the mental health services are utilised by Indigenous people within a specific sample or region [50, 51]. However, such quantification of mental health services in these studies does not include spatially explicit measures of mental health service accessibility for Indigenous people, leaving disparities in access to mental health services for Indigenous populations not being fully explored [50, 51]. Details of the evidence are presented as follows.

Geographical measures of health service accessibility in existing research mainly apply three typical methods [52]: (1) statistical index methods (i.e., measuring provider-to-population ratio for areal units); (2) spatial proximity methods (i.e., measuring travel cost, such as travel time, travel distance, or financial cost for accessing service providers); and (3) spatial interaction methods (i.e., considering the force of both supply and demand for health services in measuring the accessibility). Statistical index methods measure health service accessibility as supply per capita within a certain geographical unit for which the supply can be captured as the health practitioners (e.g., full-time-equivalent psychiatrists and psychologists [53]) and specific services consumed (e.g., referrals to a mental clinics [51]). The geographical units could be administrative areas (e.g., counties, postcode areas, suburbs, or census tracks) and zones within certain distances from a healthcare facility (e.g., a mental hospital). Statistical index methods have been favoured by governments and health organisations, with advantages of being readily implemented and being presented in absolute units, which makes these measures easily understandable and communicable to policymakers [54]. Nevertheless, this approach does not provide insights into the intricate spatial variances within a geographical unit, such as a county; the measuring result can also vary among different spatial scales applied such as postcode areas and suburbs.

Spatial proximity methods examine the minimum travel distance or time needed to access the nearest health service from residential locations [20], the average travel distance or time to all or selected health services, or the quantity of health services that can be reached within a specified distance that patients can accept [55]. Distances measured with road networks are found to be more precise than Cartesian distances (i.e., Euclidean or Manhattan distance) for measuring health accessibility [56]. Furthermore, population-weighted centroids of spatial units as origins or destinations in measuring distances are recognised as more accurate than geographical centroids in the accessibility assessment. Modes of travel are suggested to be considered in the proximity measures as access to transportation option influences accessibility to healthcare [57]. However, spatial proximity methods assume that residents will always access the nearest health service provider once seeking healthcare service, which is not realistic as people could access a provider further away from home to meet their specific needs. This type of methods also lacks consideration of both the supply and demand of health services as well as the spatial connection between them, which are essential for reflecting accessibility on the ground.

Spatial interaction methods have been developed based on the gravity model which is used to evaluate the possible interaction between any point in the population and health service providers (e.g., general practitioner) [58]. Approaches of this type, in essence, measure the possible interaction as a gravitational pull between the origin and destination, assuming this pull weakens as the spatial impedance (i.e., travel distance or time) between them increases; this pull strengthens as the demand at the origin grows or as the destination offers greater supply or becomes more attractive [59]. Radke and Mu proposed a spatial decomposition method to measure both service supply and demand within a catchment area for accessibility [60], and Luo and Wang modified the approach to a Two-Step Floating Catchment Area (2SFCA) method [61]. Since then, a number of modifications have been made to more accurately measure the accessibility. To address the issue where access within a catchment was considered uniform in the 2SFCA method, enhanced approaches (i.e., E2SFCA) employed a distance decay effect [62]. Based on the distance decay effect, Wan et al. further integrated an impedance-based selection weight in a 3SFCA method [63], and Delamater used relative distances for moderating access, to minimise the overestimation of healthcare demand in the E2SFCA method [64]. Considering that a constant catchment size could be arbitrary for the whole study area in the 2SFCA method, Luo and Whippo applied variable catchment sizes using baselines of population and provider-to-population ratio [65], whereas McGrail and Humphreys adopted a population’s remoteness to contextually implement suitable catchment sizes for urban and rural regions [66]. Further, advanced accessibility measures were developed to consider more characteristics of the access and supply of health services, such as for multiple travel modes (e.g., public transit, private car, and walking) [67, 68] and for hierarchical healthcare facilities [68, 69]. Wang introduced the inverted 2SFCA (i2SFCA) method for assessing potential crowding at healthcare facilities [70] and integrated it with the 2SFCA method into one accessibility modelling framework, going beyond solely considering resident-based accessibility in addressing the geographical variability of health service allocation [71].

Emerging efforts have been made to apply various spatial methods to measure accessibility to mental health services and accessibility to general healthcare for population sub-groups. Using a statistical index method, Cummings et al. assessed the access to mental healthcare for high- and low-income communities [25]. Regarding the spatial proximity approach, Mennis et al. used Euclidean distances to closest mental health facility [72], whereas Stulz et al. adopted road network distances to measure accessibility to mental health services [20]. Ngui and Vanasse applied a 2SFCA method to explore access to mental healthcare facilities and identify areas with service shortages [21]. More recently, Tadmon and Bearman employed the 3SFCA method to measure accessibility to psychiatrists and psychotherapists, identifying a misalignment between mental healthcare access and the need in the USA [27]. In addition, Ghorbanzadeh et al. used a measure of population-weighted travel time to the closest facility to examine mental healthcare accessibility for various age groups at the county level, focusing on seniors as a vulnerable group that may require more mental health services [22]. Jin et al. utilised a Gaussian-based 2SFCA method to assess the accessibility to multi-tier health facilities for different income groups in China, using the average housing price (i.e., RMB per square metre) in each kilometre grid as a proxy of income levels when mapping income groups [73]. Nevertheless, geographical accessibility measures, along with the advancements, are yet to be applied to measuring mental health accessibility for Indigenous populations.

On the other hand, our review of the literature shows that nine studies draw on quantitative data to comprehend and depict the patterns of Indigenous individuals’ utilisation of mental health services. These studies use service utilisation (e.g., usage rates) as realised access to indicate the access to mental health services for Indigenous people [4, 19]. While variations exist in these measures regarding types of mental health services, population coverage, and analytical scales used [4, 19, 39], further exploration is required to understand the spatial disparity of access to mental health services for Indigenous people [29]. Clark et al. tested how the facilitation for Mãori young people in New Zealand to use free counselling support influenced the mental health service access, wherein the access was measured as the ratio of individuals with a mental health referral among all the participants recruited [16]. Similar access measures were employed in studies that examined the impact of interventions or changes to improve utilisation of a particular mental health provider [4] or a mental healthcare program [19]. To explore factors that reduce access to specialist mental health services in Australia, Amos et al. adopted an access measure as a utilisation-to-population ratio (e.g., annual mental health bed days per 10,000 capita) within a large geographical unit (i.e., Statistical Area Level 3) [74]. These Indigenous-focused studies may have different measures of access depending on how utilisation was defined (e.g., for the general or specific mental healthcare) and usually compare the result with the non-Indigenous population [50, 51, 75]. However, the utilisation data does not capture those who have mental health disorders but are not engaged with relevant services. Identifying these un-served Indigenous people is important to ensure equal access to mental health services by all Indigenous populations [76]. In addition, studies focusing on specific mental health programs, initiatives, and clients may omit other types of mental health services that are available to Indigenous people [35]. Also, these studies usually emphasised on how mental health services were delivered by providers and did not consider how patients travelled to access health facilities, which could also influence the accessibility for Indigenous people [35]. Furthermore, utilisation data typically exhibits limited geographical and population coverage and is at a scale too large to reveal local disparities [74]. Consequently, measures of accessibility to mental health services for Indigenous populations often lack a geographical dimension and contribute little to our understanding of the spatial disparities in potential access within a given region.

In summary, quantitative measures of accessibility to health services have evolved progressively over the past 50 years. However, studies on mental health service access are limited to statistical index and spatial proximity methods, with a few explorations of spatial interaction methods. Advanced spatial interaction methods have been less applied in measuring mental health service accessibility, considering realistic factors such as mental health service quality (i.e., relevant workforce types). In addition, most geographical accessibility measures are for general population only, with a few studies focusing on population sub-groups, excluding Indigenous populations. Against the backdrop of developing measures for geographical accessibility to health services, there is a scarcity of applications of such measures for access to mental health services by Indigenous populations. Existing studies on the mental healthcare access for Indigenous people lack a geographical dimension and fail to capture potential accessibility, as they rely on service utilisation data. Considering the advanced spatial approaches available in measuring accessibility to health services, future research needs to develop measures that quantify the spatially explicit accessibility to mental health services for Indigenous people.

### Measures of Accessibility to Mental Health Services Is Challenged by the Diverse Range of Services Available to Indigenous People

Part of the reason for the lack of studies looking into Indigenous mental healthcare accessibility could be attributed to the diverse range of mental health services provided for Indigenous people, making the collection of data to quantify the service supply challenging. A wide range of mental health services are offered to people depending on where they live (country, state, or region) and the healthcare systems in operation. Types of mental health services can be distinguished by service contents [51], which may include psychotherapy or counselling (e.g., talking to a trained mental health professional), medication management (e.g., prescribing and monitoring of medications for mental health conditions), crisis intervention (e.g., immediate support and intervention for individuals experiencing a mental health crisis, such as severe distress), and rehabilitation services (e.g., support individuals with mental health conditions in their recovery and help them develop skills to manage daily life activities).

In Australia and other countries with a similar medical system, the mental health services can be categorised at three levels. (1) *Primary mental health services*, the first point of contact for individuals seeking mental health support, provide general mental healthcare and interventions for common mental health issues. Primary mental healthcare providers are at the community level and are mainly *General Practitioners* (GPs) who offer services typically at medical clinics, community health centres, medical centres, online or phone-based sessions, and sometimes at hospitals [77]. (2) *Secondary mental health services* are designed to provide more specialised and intensive support for individuals with moderate to severe mental health conditions, involving more specialised assessment, treatment, and care. Secondary mental healthcare providers are psychologists, psychiatrists, mental health nurses, community support workers, social workers, occupational therapists, psychotherapists, and counsellors, who offer services at mental health clinics, counselling centres, hospitals, community mental centres, or specialised treatment centres [78]. (3) *Tertiary mental health services* are the highest level of specialised care, typically provided for individuals with severe and complex mental health conditions that often require long-term management and specialised interventions. Tertiary mental healthcare providers are usually teams of health practitioners that also provide secondary mental health services [79], and the facilities for tertiary mental health services include inpatient psychiatric hospitals, specialised clinics and centres, forensic hospitals and correctional facilities, rehabilitation centres and facilities, and so forth [80]. It should be noted that GPs working as the first point of contact to provide mental healthcare are only required for Medicare-subsidised sessions of the secondary and tertiary mental health services. If a person is seeking secondary or tertiary care through private practice without a referral from a GP, the person will pay for the sessions out of pocket or through their health insurance.

In addition, mental health services can be funded by both public and private sectors. A country may have a complex funding system for delivering mental health services. For instance, in Australia, Medicare-funded mental health services are available to all Australian including Indigenous people through the Medicare Benefits Scheme [81] while the waiting time to receive healthcare could be longer than that in the private practices; other government-funded mental health programs include the Indigenous Mental Health First Aid program and the Indigenous Suicide Prevention program [82]. Nonetheless, mental health services provided by private practices are neither free nor low cost for Indigenous people, even when the service is in the closest proximity, potentially making the service inaccessible for them. Some accessibility studies discussed strategies to enhance access to mainstream mental health services for Indigenous people but did not specify how the services would be funded [17, 44].

Measuring geographical accessibility to mental health services needs to consider service types, especially in relation to service locations and availability. Online mental health services have been gaining popularity, especially since the COVID-19 pandemic [76, 83], and measuring accessibility to such services would be different from that of traditional therapies that require physical access. Most studies on health service accessibility use the number of health facilities (e.g., medical clinics and hospitals) within certain geographical units (e.g., postal zones) to measure health service supply [21, 69]. However, facilities on different levels or even the same level could have various capacities of providing particular health services (e.g., mental health), where service counts as fulltime equivalent could be a more accurate alternative measure [67]. In addition, the mental health service types available for Indigenous people include both mainstream services via Medicare Benefits Scheme and special mental health programs exclusive to Indigenous populations, which needs to be taken into account when measuring accessibility to mental health services for Indigenous people.

### Limited Understanding of Barriers for Indigenous People to Access Mental Health Services

To quantify access to mental health services by Indigenous people, it is important to also understand the service demand and how Indigenous individuals gain access to the services. For Indigenous population, understanding the barriers that hinder their access to mental health services as needed is the critical first step to fully quantify their service demand and measure the accessibility for them. On the one hand, geographical isolation or remoteness and socioeconomic disadvantages are significant barriers for Indigenous populations [45, 74]. Though no previous studies have taken into account these factors for measuring mental health service accessibility for Indigenous people, some efforts have been made to consider remoteness in measuring a nationwide accessibility to primary health services [66], and mapping health service accessibility for different income [25] and age groups [22], as mentioned in “Measures of Accessibility to Mental Health Services Is Challenged by the Diverse Range of Services Available to Indigenous People” section. On the other hand, a lack of reliable transportation could make it difficult for Indigenous people to access mental health facilities [84]. Measures of primary healthcare accessibility have considered this barrier by accounting for travel cost (e.g., travel time) by multiple travel modes (e.g., public transport and driving) [67]. Other barriers that hinder Indigenous people’s access to mental health services are discussed below, which also need to be considered in future research to quantify mental health accessibility by Indigenous people.

Lack of culturally appropriate services and trust in mainstream health services is an important barrier for Indigenous people to access services and effectively address their mental health needs [85], as they perceive mental health and well-being differently due to the cultural and language differences [15, 44]. Specifically, cultural continuity has been identified as a determinant of Indigenous populations’ health [86]. The effects of colonialism, neo-colonialism, racism, and other factors vary across Indigenous populations and communities, making the mental health needs of Indigenous communities differ from each other [15]. A lack of culturally appropriate care can lead to decreased motivation for accessing mental health services and increased reliance on informal support systems (e.g., family members, friends, or other informal networks) [34]. In addition, Indigenous populations have endured historical trauma and discrimination, leading to a stigma surrounding mental health issues [44, 87], a lack of trust in mainstream services, and thus challenging access to mental health services [34].

Another barrier encountered by Indigenous people to access mental health services is the lack of familiarity with mainstream mental health services offered within their local area and limited early intervention supports. Indigenous individuals may hold the belief that the only way to seek support from mainstream mental health services is by presenting at Emergency Departments during times of crisis or precarious situations where they fail to be taken seriously or receive assistance [17]. This barrier is related to the insufficient mental health literacy which encompasses the capacity to recognize mental illness and access suitable treatment options [18]. To address this barrier, central coordination plays an important role in aiding in referral coordination, facilitating Indigenous people’s access to mental health support, and improving the communication and feedback between and within services [88, 89]. As such, the need of central coordination in mental healthcare pathways has been highlighted [90].

Access to mental health services for Indigenous populations can be limited due to barriers such as a lack of culturally appropriate services and a lack of awareness about available services. Though these barriers have been highlighted in some qualitative studies on Indigenous people’s access to mental health services [44, 47], less attention has been paid to consider these barriers and relevant needs of Indigenous populations in measuring geographical accessibility to mental health services for them.

## Discussion

This study systematically reviews existing peer-reviewed literature on accessibility to mental health services for Indigenous populations, highlighting the methods and considerations employed incorporating and gauging geographical accessibility to mental health services for population sub-groups. Findings show that a relatively small number of studies have focused on Indigenous mental health service access, and most of these studies employed qualitative methods using interview or survey data [17, 41]. Limited studies have quantified Indigenous populations’ access to mental health services, and mostly at the scale of countries or states [19, 74].

Qualitative studies focus on conceptualising access to mental health services as a pre-treatment process before receiving mental healthcare including multiple stages from recognising the problem to contacting the selected service [44, 47]. This process of access to mental health services is specified for Indigenous groups by considering individual characteristics such as socio-demographics [40, 42] and marginalised groups (e.g., gender minority) [45, 46], which highlights Indigenous people’s different situations for accessing mental health services [15]. This represents the dynamic and complex nature of factors that influence Indigenous individuals’ decision to access and use mental health services, which could be comprehensively considered in future research measuring accessibility to mental health services for Indigenous people.

Quantitative approaches to measure accessibility to health services (e.g., primary healthcare) are available but not applied to mental health services for Indigenous people. Various methods are used to quantify health service accessibility [52, 71] and include statistical index (e.g., provider-to-population ratios) [53, 91], spatial proximity (e.g., minimum travel time to access the closest health service) [20, 55], and spatial interaction approaches to consider locations of service providers, locations of users, and travel impedance in between (e.g., gravity model, 2SFCA, E2SFCA, 3SFCA, and i2SFCA) [61, 65, 71]. A growing research interest concentrates on developing more sophisticated measures to capture health service access more accurately by considering multiple travel modes [67] and hierarchical healthcare facilities [69]. Limited attempts have been made to use spatial interaction methods to measure geographical accessibility to mental health services [21], to quantify the potential for mental health services (e.g., using pertinent workforce data) in accessibility assessment [27], and to differentiate geographical accessibility measures for some sub-groups in the population such as age and income groups [25, 73]. Nevertheless, most of these geographical accessibility measures, especially those using spatial interaction approaches, have yet to be applied for Indigenous people. In addition, though some studies quantitatively explored Indigenous people’s access to mental healthcare, they employed data on utilisation or uptake of general or specific mental health services [4, 19]. These studies (1) failed to capture the demand to include those who suffered from mental health disorders but not accessing relevant health services [19]; (2) lacked consideration of individual details such as how a person travels to access services [35]; and (3) had limited geographical and population coverage and used geographical scales at a coarse level that are not applicable to identify local disparities [74]. In sum, spatially explicit accessibility to potential mental health services for Indigenous populations is yet to be measured, and local spatial disparities in access remain unexamined.

The lack of empirical studies exploring the accessibility issue for Indigenous people could be partially due to the diversity in mental health services making it difficult to collect data that quantifies the service supply. Types of mental health services can be distinguished by service contents, levels of healthcare corresponding to mental health conditions, and funding resources from public or private sectors, particularly with regards to service locations and availability [51]. Sourcing data on different types of mental health services, especially information regarding their location and availability, presents a challenge in measuring geographical accessibility to mental health services for Indigenous people. As mental health services at multiple healthcare levels could be delivered at the same facility, and different facilities could have various capacities, using the number of facilities to quantify service supply in accessibility measures is subject to bias [21]. Medicare-funded mental health services and special mental health programs (e.g., Indigenous medical clinics) are both available to Indigenous people, though the former is shared with the regional population while the latter is exclusive to Indigenous communities. There are also government-funded programs such as the Mental Health Services in Rural and Remote Areas program in Australia [92] which could be preferred in mental health service selection of Indigenous people. Future measures of mental health service accessibility may take the diverse range of service types into consideration as it could influence the priority of Indigenous people to select mental health services for access.

To quantify the service demand for the Indigenous population and develop specialized mental health service accessibility measures, it is essential to fully comprehend the obstacles that impede their access to mental health services. Various barriers that hinder Indigenous people to access mental healthcare include geographical isolation along with transportation issue, a lack of culturally appropriate services, a lack of trust in mainstream services, and unawareness of available mental health services in locales. Some barriers (e.g., remoteness, socioeconomic disadvantages, transportation issue) have been considered in measuring the accessibility to primary health services [22, 65–67]. Other barriers (e.g., historical trauma and unawareness) have not been considered in accessibility measurement but reflects Indigenous peoples’ needs different from their non-Indigenous counterparts [17]. This consideration can be integrated into future accessibility measures with enhanced service provision and delivery. For example, Indigenous medical or health services as culturally appropriate services could be assigned with higher weights in quantifying service supply of accessibility as they are more reliable for and attractive to Indigenous people than general primary healthcare [63]. For rural or remote areas where sparse population cannot support mental healthcare resource allocation at an urban level [93], it may be beneficial to advance variable catchment sizes and distance decay functions in E2SFCA-based methods. This adjustment can better accommodate the service density difference between rural and urban areas for Indigenous people, aiding in measuring accessibility and preventing bias in distributional inequality [94].

To the best of our knowledge, this review represents the first literature synthesis to examine the evidence on how accessibility to mental health services for Indigenous populations is understood and measured worldwide. This review highlights research gaps wherein (1) the comprehensive understanding of Indigenous people’s access to mental health services has not been fully applied in quantitatively measuring their mental healthcare accessibility. (2) Existing quantitative measures have not gone beyond service utilisation rates or provider-to-population ratios; they approached the geographical access to mental health services for Indigenous people without a finer scale to consider individual details such as how people travel to health facilities. (3) Diversity of mental health service types and various barriers experienced by Indigenous people reflect their divergent patterns of using mental health services, of which few have been fully considered and embedded in measures of geographical accessibility to mental healthcare. One possible reason for these gaps could be a lack of standardised accessible data at a fine resolution that helps capturing details of mental health service providers and users, impedances of the accessing process (e.g., travel cost), and relevant locational information [29]. Future research can enrich data resources by collaborating with regulators and governments in data collection, drawing on advanced methods in measuring geographical accessibility to healthcare services, and developing accessibility measures integrating understanding of how Indigenous individuals use mental healthcare with respect to service diversity, barriers, and individual characteristics [87].

There are two limitations in this study. First, the lack of suitable quality assessment tools for evaluating the methodological rigor of quantitative research could potentially affect the level of reliability in assessing systematic errors. Second, though a systematic review methodology is sturdy, its effectiveness is constrained when it comes to developing implications for intricate problems like modifying geographical measures of accessibility using factors identified in qualitative research (e.g., individuals making selection in potential mental health services for access). In addition, the limited number of articles studying accessibility to mental health services for Indigenous populations could also result in limited implications derived from these studies.

## Conclusion

Qualitative and quantitative measures of accessibility to mental health services for Indigenous populations play a vital role in tackling ongoing Indigenous mental health disparities, guiding allocation of health resources, and supporting policymaking grounded in evidence. This review reveals that existing research has employed qualitative approaches to understand the access to and utilisation of mental health services for Indigenous populations in order to achieve better mental health outcomes. Despite the existence of spatially explicit methods developed to quantify potential access to health services in general, there is limited application of such methods in measuring accessibility to gain understanding of how Indigenous individuals use the diverse range of mental health services and the barriers they have in accessing these services. This lack of understanding makes it challenging to apply the geographical methods to quantify accessibility of mental health services for Indigenous populations. The scarcity of data that is available and applicable for measurement has also contributed to this challenge. Built upon the findings of this review, future research endeavours could investigate mental health service resources available to Indigenous people and culturally appropriate service needs within Indigenous communities. By gathering empirical data and developing spatially explicit methodologies at a finer scale, it is possible to advance the quantitative measures of mental health service accessibility for Indigenous populations and better inform policymaking to enhance Indigenous people’s access to mental healthcare.

## Appendix

Table 1

**Table 1:** Twenty-seven studies published in chorological order that were selected for inclusion in our systematic review

Author(s), year	Research method	Measure of accessibility	Description of accessibility/access to mental health services for Indigenous people
Takeuchi, Mokuau, and Chun (1992) [38]	Critical analysis and intervention review	Qualitative	Availability of mental health services provided within the targeted ethnic community, with which the service hours are extended into evenings and weekends for ethnic groups and walk-in appointments are accepted
Fuller, Edwards, Procter, and Moss (2000) [48]	Semi-structured in-depth interviews and thematic analysis	Qualitative	Mental health help-seeking pathway that people use from identifying the symptoms of mental health illnesses to using mental health services in rural and remote communities
Yeh et al. (2002) [51]	Logistic regressions	Quantitative	Utilisation of public outpatient mental health services by young individuals from different racial/ethnic groups, presented in three aspects including patterns of referral, diagnosis, and types of services and captured as categorical features of individual patients
Willging, Salvador, and Kano (2006) [45]	Semi-structured yarning interviews and thematic analysis	Qualitative	Mental health help-seeking process which is a sequence of practical choices shaped by local viewpoints on mental health, social connections, factors within the treatment system, and other contextual aspects of rural communities
Stathis et al. (2007) [75]	Descriptive statistics and retrospective analysis using chi-squared analysis	Quantitative	Rates of Indigenous and non-Indigenous young people who had been referred and subsequently received a mental health service to the total Indigenous and non-Indigenous young people admitted into detention, respectively
Denman (2007) [46]	Consultations with key stakeholders and thematic analysis	Qualitative	Mental health help-seeking process of attempting to access public mental health services for deaf individuals from an Indigenous Australian diverse background, which is influenced by barriers based on communication or cultural diversity
Isaacs, Pyett, Oakley-Browne, Gruis, and Waples-Crowe (2010) [44]	Conceptualisation and critical analysis	Qualitative	Mental health help-seeking process defined as a sequence of four successive steps: recognising the existence of an issue, determining the necessity of treatment for resolution, choosing to seek treatment, and contacting the mental health service
Williamson et al. (2010) [42]	Semi-structured focus group interviews and thematic analysis	Qualitative	Availability of mental health services to young people, impacted by waiting time exceeding 1 year and perceived potential of incurring unnecessary intervention from government organisations
Zinck and Marmion (2011) [41]	Critical analysis	Qualitative	Availability of mental health services for Indigenous populations featured with proximity at the community scale, mental health concepts by Indigenous people, traditional healing combined with conventional mental health services, and cultural congruence
Isaacs, Maybery, and Gruis (2012) [43]	Semi-structured interviews, focus group discussions, and thematic analysis	Qualitative	Mental health help-seeking process defined as a sequence of four successive steps: recognising the existence of an issue, determining the necessity of treatment for resolution, choosing to seek treatment, and contacting mental health services; Aboriginal people have more difficulties in the final step than non-Aboriginal people, being related to entering services, engagement with services, and staffing problems
Koopmans, Uiters, Deville, and Foets (2013) [50]	Multilevel logistic regression	Quantitative	Utilisation of outpatient mental health services, assessed by asking respondents whether any interaction had occurred in the preceding 12 months with mental health services (i.e., mental health care centres, psychiatric outpatient clinics, alcohol or drug-abuse clinics, psychiatric emergency centres, independent psychiatrists, psychologists, psychotherapists, sexologists, or other psychosocial treatment facilities)
Price and Dalgleish (2013) [34]	Focus group interviews and thematic analysis	Qualitative	Availability of mental health services embedded in help-seeking behaviours among Indigenous young people, influenced by their motivations and barriers to using formal support services, preferences for different help-seeking modalities (e.g., phone, email, and real-time web), and awareness and perceptions of youth counselling services
Clark et al. (2014) [16]	A quasi-experimental pre-/post-intervention design with a series of questionnaires and descriptive statistics	Quantitative	Utilisation of interventions to facilitate Indigenous young people with mild to moderate mental health disorders to access appropriate mental healthcare, evaluated by the proportion of patients choosing to start the intervention in all patients who received the referral about the intervention during a period of 2 years
Panaretto, Wenitong, Button, and Ring (2014) [39]	Descriptive statistics and critical analysis	Quantitative	Utilisation of Aboriginal Community Controlled Health Services (ACCHSs), captured as the number of recorded visits by Aboriginal and Torres Strait Islander patients within 2 years
Hepworth et al. (2015) [4]	Descriptive statistics, open-ended interviews, and thematic analysis	Quantitative	Utilisation of mental healthcare provided by a psychologist and a social worker included as core members of a primary health care team, captured as the number of the cumulative instances of service provided by the social worker and psychologist, the count of referrals to the psychologist initiated by general practitioners (GPs), and the number of general practice mental health care plans within 2 years
Reifels et al. (2015) [19]	Descriptive statistics, regression analysis and *t* test	Quantitative	Utilisation of Indigenous Access to Allied Psychological Services (ATAPS) and mental healthcare provided to Indigenous Australians via other ATAPS initiatives, captured as the number and proportion of Indigenous client referrals made to Indigenous ATAPS services and all other ATAPS initiatives for each Medical Local within 10 years
Rachael, David, Lesley, Richard, and Tricia (2015) [36]	Key informant interviews within a participatory action research framework and thematic analysis	Qualitative	Availability of mental healthcare via appropriate pathways including early intervention, assessment, and treatment of depression within the Indigenous community, with particular attention to four targeted groups: young individuals, perinatal women, individuals dealing with chronic disease, and healthy adults
Browne et al. (2016) [37]	In-depth interviews and interpretative thematic analysis	Qualitative	Availability of equity-oriented mental healthcare for Indigenous people, as their experiences of healthcare are influenced by structural violence including traumatic, negative impacts of historical and ongoing colonialism, discrimination in health care, and lack of consideration of Indigenous people’s specific needs for health services
Tenenbaum and Singer (2018) [14]	Narrative inquiries including questionnaires, interviews, and analysis using a narrative story map tool	Qualitative	Availability of mental health care services for Indigenous Latinx gender-fluid border-youth with their specific needs including culturally informed counselling service (e.g., services in Indigenous native languages), consideration of their precarious immigrant legal status, and cultural patterns of gender development for them
Horrill, McMillan, Schultz, and Thompson (2018) [49]	Critical analysis and postcolonial analysis	Qualitative	From a biomedical perspective, the measure is physical accessibility to mental health services that is associated with geographical distance, the presence of services and healthcare providers, as well as the financial capacity to overcome geographical barriers to access services, with the assumptions of (1) individuals’ awareness of the services; (2) underlying individual choice and responsibility to access mental health services; and (3) those services are equally accessible for all From a postcolonial perspective, based on service availability captured in the physical accessibility, the service delivery is emphasised to equally consider the influence of contextual factors, and historical along with social barriers that must be overcome by patients (e.g., structural disadvantages that impact Indigenous peoples’ opportunities and health conditions)
Titov, Schofield, Staples, Dear, and Nielssen (2019) [76]	Treatment outcome comparison using ANOVA and chi-squared analysis	Quantitative	Number of online mental health service units provided at MindSpot for Indigenous respondents, including free assessment and seven treatment courses, namely, the Wellbeing Course, Wellbeing Plus for older adults, Mood Mechanic for younger adults, the Indigenous Wellbeing Course, and courses for the treatment of post-traumatic stress disorder, obsessive compulsive disorder, and a course treating the disability and distress associated with chronic pain
Deanna et al. (2019) [40]	Semi-structured interviews and thematic analysis	Qualitative	Pathways to mental health care provided by Aboriginal Community Controlled Health Services (ACCHSs) for urban Aboriginal young people, considering mental health specialist available in-house at the ACCHS and the role of dedicated child and adolescent Aboriginal Health Workers (AHWs) (e.g., Bridging the communication gap, providing support and transport, and facilitating care alongside mental health specialists)
Boksa et al. (2021) [35]	A questionnaire survey and descriptive analysis	Qualitative	Availability of mental health services from the ACCESS Open Minds (AOM)^a^ research network at sites of Indigenous communities for youth aged 11–29, with a focus on the demographic and clinical portrait of Indigenous youth presenting at mental health services
Nolan-Isles et al. (2021) [47]	Semi-structured interviews and thematic analysis	Qualitative	Availability of mental health services for Aboriginal people living in regional and remote Australia, conditioned by factors at national (e.g., funding), state (e.g., travel and accommodation subsidies), and community (e.g., reliable and affordable services, and transport availability) levels
Amos, Coleman, Spring Walsh, and Gardiner (2022) [74]	Retrospective analysis using ANOVA and a multivariate log–log regression	Quantitative	Measure 1—annual admissions per 10k capita: The number of admissions within 1 year to public specialist (inpatient) mental health units by residents living in the SA3 per 10k people resident in that SA3 Measure 2—annual mental health bed days per 10k capita: The number of overnight bed days within 1 year in public specialist (inpatient) mental health units by residents living in the SA3 per 10k people resident in that SA3. This measure includes partial admissions where one but not both the admission and discharge date was outside the target period
Smye, Browne, Josewski, Keith, and Mussell (2023) [15]	Interviews and an interpretive thematic analysis	Qualitative	Availability of mental health and/or substance use services from selected Indigenous community controlled mental healthcare agencies and primary healthcare providers in an inner-city area, considering normalisation of social distress, recreation of trauma, the challenge of harmonising restricted lives with harm reduction, and alleviating suffering through relational practice
Garay et al. (2023) [17]	Semi-structured yarning interviews and thematic analysis	Qualitative	Availability of mainstream mental health services for aboriginal young people in one metropolitan and one inner-regional region, considering early intervention services targeting Aboriginal young people’s Social and Emotional Wellbeing (SEWB), service availability for Aboriginal young people, supportive relationships with local Aboriginal communities, mental health assessments at Emergency Departments (Eds), and SEWB support of Aboriginal Community Controlled Health Services (ACCHSs) for Aboriginal young people

^a^The purpose of the AOM network is to execute and systematically assess a reformation of mental health services catering to individuals aged 11–25 at 14 locations throughout Canada, encompassing six Indigenous communities
